# The High Diagnostic Accuracy of Combined Test of Thyroid Transcription Factor 1 and Napsin A to Distinguish between Lung Adenocarcinoma and Squamous Cell Carcinoma: A Meta-Analysis

**DOI:** 10.1371/journal.pone.0100837

**Published:** 2014-07-08

**Authors:** Li Li, Xiaorong Li, Jieyun Yin, Xia Song, Xiaochen Chen, Jiane Feng, Hongyu Gao, Li Liu, Sheng Wei

**Affiliations:** 1 Department of Epidemiology and Biostatistics, School of Public Health, Tongji Medical College of Huazhong University of Science and Technology, Wuhan, Hubei, China; 2 Deptartment of Cardiovascular Surgery, Union hospital, Tongji Medical College of Huazhong University of Science and Technology, Wuhan, Hubei, China; 3 Renal Division, Tongji Hospital, Tongji Medical College of Huazhong University of Science and Technology, Wuhan, Hubei, China; Univesity of Texas Southwestern Medical Center at Dallas, United States of America

## Abstract

**Background:**

Accurate classification of non-small cell lung cancer (NSCLC) using morphological features has several limitations. However, the use of thyroid transcription factor 1 (TTF-1) and Napsin A as markers for the identification of various subtypes of NSCLC has shown promise. This meta-analysis was designed to evaluate the diagnostic value of combined TTF-1 and Napsin A test to distinguish lung adenocarcinoma from squamous cell carcinoma.

**Methods:**

The Medline, EMBASE and Web of Science databases were searched, along with the reference lists of relevant articles (up to May 4, 2014). Ten studies containing 1,446 subjects were identified. The sensitivity, specificity, diagnostic odds ratio (DOR) and area under the summary receiver operating characteristics curve (AUC) were calculated to estimate the combined diagnostic value of TTF-1 and Napsin A.

**Results:**

The pooled sensitivity and specificity were 0.76 (95% CI: 0.69–0.83) and 1.00 (95% CI: 0.92–1.00), respectively. The positive and negative likelihood ratios were 877.60 (95% CI: 8.40–91533.40) and 0.24 (95% CI: 0.18–0.32). The DOR was 3719 (95% CI: 33–414884). The AUC was 0.92 (95%CI: 0.89–0.94). The patient's location was a source of heterogeneity for sensitivity. The patient's location, the study's sample size and the threshold used to determine positive staining were consistently found to be sources of heterogeneity for specificity in subgroup analyses and meta-regression.

**Conclusions:**

The combined test of TTF-1 and Napsin A presents a promising alternative method, useful to distinguish between lung adenocarcinoma and squamous cell carcinoma.

## Introduction

Lung cancer is the leading cause of cancer mortality worldwide. Approximately eighty percent of lung cancers are determined to be non-small cell lung cancer (NSCLC), a heterogeneous group comprised of adenocarcinoma (AC) and squamous cell carcinoma (SQCC) [1]. Due to the prevalence of NSCLC, extensive researches in the field have enabled the development of various therapies targeting specific types of lung cancer [2]. Therefore, demand for the accurate classifications of different subtypes of NSCLC has risen to allow maximization of the subsequent therapeutic response and minimization of any adverse effects, especially for poorly differentiated non-small cell lung carcinomas, which were classified generally as non-small cell carcinoma not otherwise specified (NSC NOS) based on hematoxylin-eosin (H&E) immunohistochemistry method [3]–[5]. However, poorly differentiated non-small cell lung carcinomas could be further divided into several subtypes, mostly into adenocarcinomas (AC) and squamous cell carcinoma (SQCC), which require different treatment strategies. For example, AC frequently harbour activating mutations of the epidermal growth factor receptor gene (EGFR) or EML4–ALK rearrangements, so epidermal growth factor receptor (EGFR) inhibitors and vascular endothelial growth factor (VEGF) inhibitors are more effective in treating AC than SQCC [6]–[8]. SQCC commonly express insulin-like growth factor 1 receptor (IGF-1R), which is a target for figitumumab. In addition, molecularly-targeted therapies such as Avastin should not be used to treat SQCC patients, as it has a 30% mortality rate, due to fatal hemoptysis [9], [10]. Therefore, misdiagnosis or inaccurate diagnosis of poorly differentiated non-small cell lung carcinoma by single hematoxylin-eosin (H&E) immunohistochemical method may lead to patients' insensitivity to subsequent therapies and maximization of adverse effects. And this will in turn lead to difficult management of lung cancer patients and huge consumption of medical and health resources. Alternatively, the use of new molecular biomarkers, whose identification only require a small amount of cells, has been proposed as a powerful tool to distinguish AC from SQCC [11]–[14]. The value of immunohistochemical markers has been well established in separating poorly differentiated AC from SQCC [12], [15]–[17]. Two such markers recently showing promise are Thyroid transcription factor 1 (TTF-1) and Napsin A.

TTF-1, a highly-conserved homeodomain-containing transcriptional factor involved in the early development of lung. It is a well established immunomarker for lung adenocarcinoma, with sensitivity ranging from 60% to 100%, and specificity ranging from 97% to 100% [18]–[21]. Napsin A, a functional aspartic proteinase expressed in the cytoplasm of healthy lung parenchyma, has recently been reported as a promising immunomarker associated with lung adenocarcinomas [22], [23]. And the potential of TTF-1 and Napsin A has been revealed in separating AC from SQCC even in poorly differentiated NSCLC [17]. Stoll et al. reported the sensitivity and specificity for Napsin A immunohistochemistry in diagnosing poorly differentiated pulmonary ADCs were 65% and 96%, respectively [16]. Besides the advantage in the diagnosis of lung adenocarcinoma, both TTF-1 and Napsin A have also been found in biopsies of lung squamous cell carcinoma at rates of 2% to 13% for TTF-1, and upwards of 26% for Napsin A [23]–[28]. This has led researchers to propose the combined test of TTF-1 and Napsin A in order to subclassify NSCLC [29], [30]. However, the diagnostic accuracy of the combined test has varied among studies, with the specificities ranging from 0.88 to 1.00 [22], [24], [27], [31], and the sensitivity between 0.47 and 1.00 [1], [8], [30], [32]. Until now, the diagnostic value of the combined test has not been systematically evaluated. The present meta-analysis was designed to summarize the evidence behind the value of a combined TTF-1 and Napsin A test for subtype-classification of lung AC and SQCC in NSCLC.

## Materials and Methods

### Literature search

A thorough search of Medline, EMBASE and Web of Science (up to May 4, 2014) was conducted to identify eligible studies. The following search terms were used: “lung cancer” OR “lung neoplasms” OR “lung neoplasm” OR “lung carcinoma” OR “lung tumor” OR “pulmonary neoplasm” OR “pulmonary neoplasms” OR “pulmonary cancer” OR “carcinoma, non-small cell lung” OR “non-small cell lung cancer” OR “non-small cell lung carcinoma” OR NSCLC, “thyroid transcription factor-1” OR TTF-1, Napsin A, without language restrictions. The references of any relevant articles were also scanned for potentially missing studies.

### Selection criteria

Selection criteria were given as follows: (1) the combined detection of TTF-1 and Napsin A was used to distinguish between AC and SQCC; (2) the combined test of TTF-1 and Napsin A was serial test, which mean tissues with positive immunohistochemical staining for both TTF1 and Napsin A were determined as adenocarcinomas, otherwise, as squamous cell carcinoma; (3) morphological diagnoses, such as hematoxylin-eosin staining, were used as the reference diagnostic standard; (4) results were reported in numbers of true-positive, false-positive, true-negative, and false-negative, or sufficiently detailed data were presented to derive these numbers. Studies were excluded if they were as follows: (1) a case report, review or conference proceeding; (2) containing no specific results regarding lung cancer; (3) with a sample size less than 50. For multiple or duplicate publications that covered the same dataset, only the most recent or complete study was included.

### Data extraction and quality assessment

The two investigators independently extracted data from all studies fulfilling the inclusion criteria. The following information was extracted from each study: first author, year of publication, patients' location, sample size, the number of AC and SQCC, specimen type and the threshold for staining positivity. Any disagreement was resolved by consensus. The quality of methodology for each study was assessed using the quality assessment of diagnostic accuracy studies (QUADAS) [33]. For each item, a score of 1 was applied if the answer was “yes”; otherwise, a score 0 was applied.

### Statistical analysis

For studies with true-positive, true-negative, false-positive and false-negative numbers available, we computed the following parameters and corresponding 95% confidence intervals for each study: sensitivity, specificity, and the diagnostic odds ratio combined by positive and negative likelihood ratios (LRs). Data were finally pooled in summarized receiver-operating characteristic curves (sROC), where the area under the sROC (AUC) measures test precision. The heterogeneity was explored with a Cochran's chi-square test and quantified by calculating the I^2^ statistic to reflect the degree of variability in results across studies. To assess any potential confounding factors, including the patient's location (Western vs. Asian), specimen types (resected vs. others), sample size (over 100 vs. less than 100), and threshold for staining positivity (≥1% vs. ≥10%), the subgroup analyses and meta-regression were performed taking the above factors into account. Additionally, sensitivity analysis was also performed to assess the influence of any individual study on the overall estimate.

In order to test for publication bias, Deek's funnel plot method was applied. Statistical analyses were performed using Midas module in the Stata (Version 10.0), and all *P*-values calculated as two-sided. The association was considered significant if the *P-* value was less than 0.05.

## Results

### Characteristics and methodological quality of include studies

**Figure 1:**
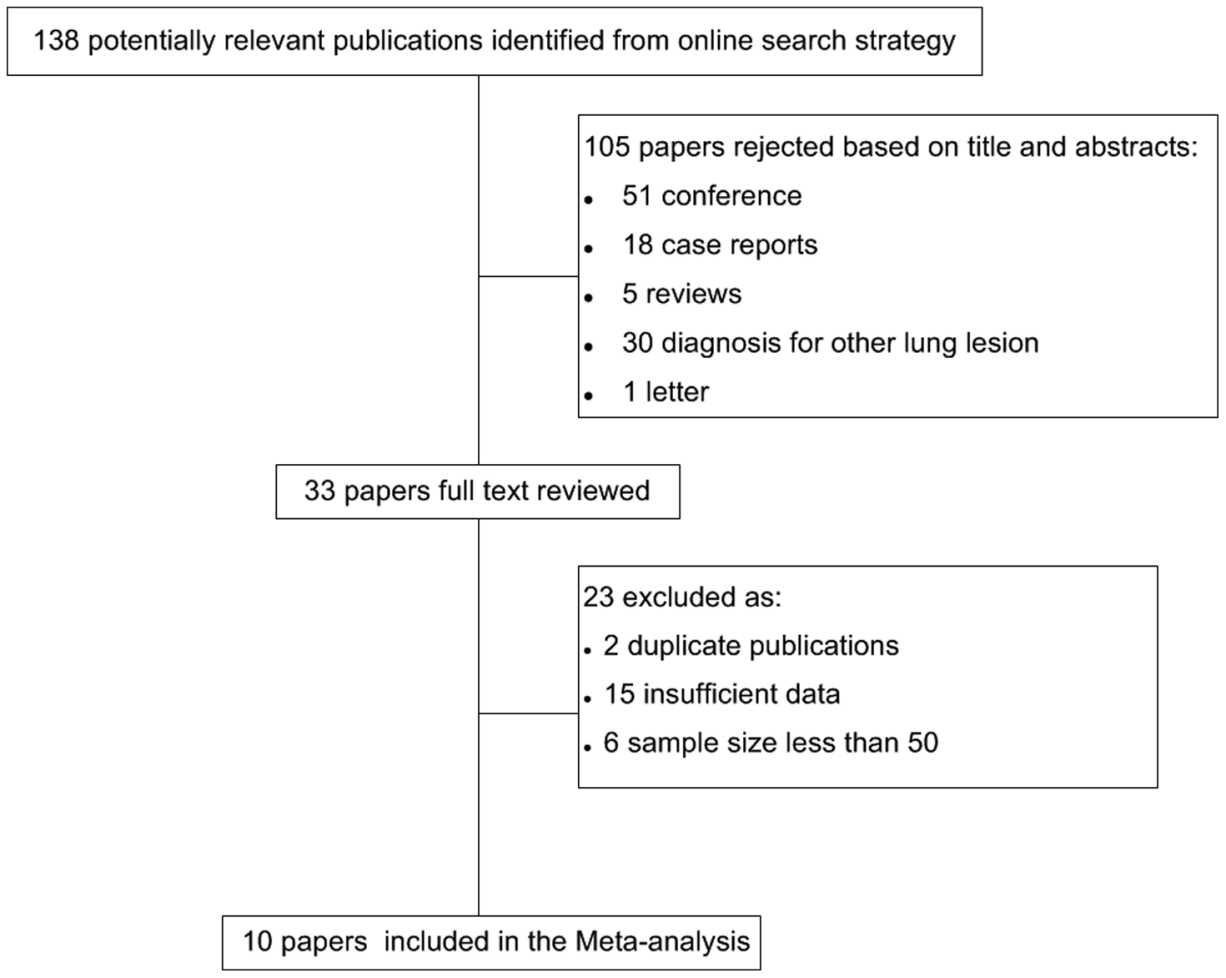
Study flow chart for the process of selecting the eligible publications.

The search of the databases produced 222 studies, of which 84 were excluded for duplication, leaving 138 potentially relevant studies to be retrieved. One hundred and five studies were excluded following the first screening based on abstracts and titles. Thirty-three studies were then retrieved for full text review. After carefully reading the full text articles, 2 were excluded for duplication and 6 studies were excluded for containing a sample size less than 50 [1], [9], [10], [12], [29], [34]. And 15 studies were excluded for containing insufficient data to calculate sensitivity and specificity. Finally, 10 studies were eligible for inclusion in this meta-analysis [8], [11], [22], [24], [27], [31], [32], [35]–[37] (Figure 1). The characteristics of the included studies are detailed in Table 1.

**Table 1:** Characteristics of Studies Included in the Meta-analysis.

First Author	Year	Country	Sample size	AC/SQCC	Specimen type	Stain positive
Bishop	2010	America	102	50/52	resected specimens	≥1%^a^
Yang	2010	America	197	158/39	resected specimens	≥1%
Zhang	2010	China	297	212/85	resected specimens	≥10%^b^
Yanagita	2011	Japan	64	39/25	surgical,biopsy cases	≥1%
Fatima	2011	America	59	35/24	FNA	≥1%
Turner	2012	Japan	188	94/94	resected specimens	≥1%
Tacha	2012	America	210	115/95	resected specimens	≥10%
Noh	2012	Korea	74	36/38	resected specimens	≥1%
Collins	2013	America	69	35/34	FNA	≥10%
Brunnström	2013	Swedish	186	121/65	resected specimens	≥1%

NOTE:

a , cases with more than 1% of tumor cells staining were classified as positive;

b , cases with more than 10% of tumor cells staining were classified positive;

FNA, Fine-needle aspiration; AC, lung adenocarcinoma; SQCC, squamous cell carcinoma.

**Figure 2:**
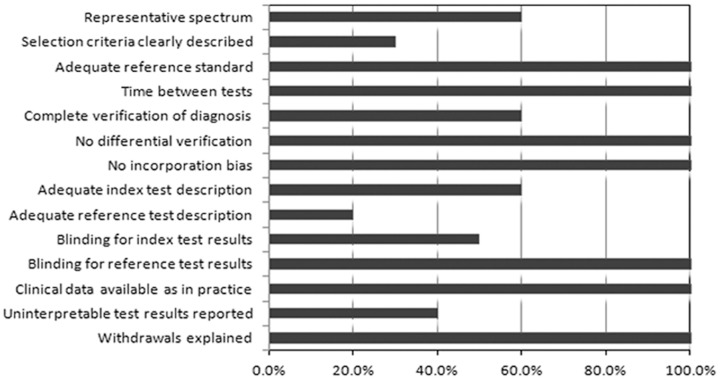
The methodological quality assessments of studies included in the meta-analysis, the vertical coordinate presents 14 QUADAS items of the quality assessment of diagnostic accuracy studies (QUADAS); the horizontal axis presents percentages of 14 QUADAS items fulfilled by studies included in the meta-analysis.

The quality assessments on the included studies are presented in Figure 2. Out of 14 QUADAS items, item 1 (spectrum composition) and 2 (selection criteria) describe the variability of the studies, while items 8 (index test execution), 9 (reference standard execution) and 13 (uninterpretable test results) assess the quality of the reporting. The remainders are about the bias of the studies. The QUADAS scores of the studies ranged from 8 to 12 with a median score of 10. Items 1 and 2 were 60.0% and 30.0% fulfilled by studies respectively, indicating high variability among studies. The execution proportions of item 8, 9 and 13 were 60.0%, 20.0% and 40.0% respectively, suggesting poor reporting quality of studies. The remainders of the items reached a level of 100%, with exception of items 5 (verification of diagnosis), and 10 (blinding for index test results), which were only 60.0% and 50.0% fulfilled by the studies, respectively.

### Meta-analysis and heterogeneity

**Figure 3:**
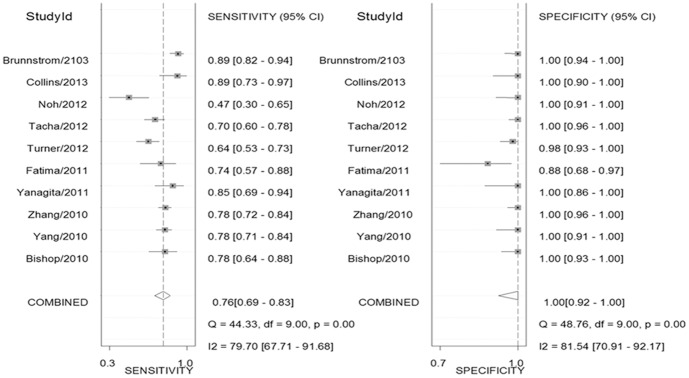
Pooled sensitivities and specificities of combined test of TTF-1 and Napsin A in distinction between AC and SQCC.

**Figure 4:**
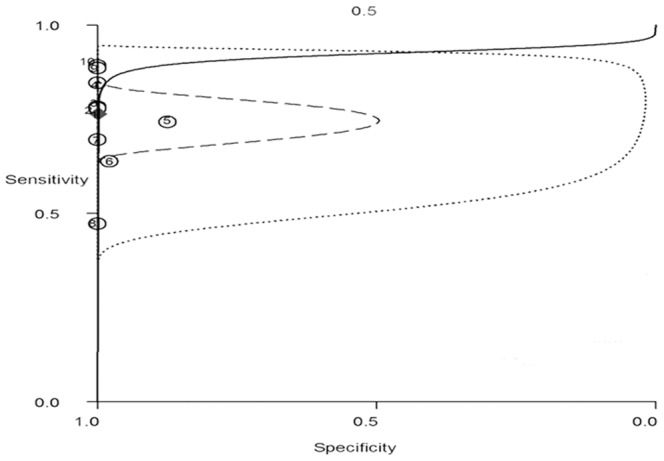
ROC curve analyses of combined test of TTF-1 and Napsin A in distinction between AC from SQCC. ACU = 0.92[0.89–0.94], sensitivity = 0.76[0.69–0.83], specificity = 1.00[0.92–1.00]. Solid line presents sROC; circle presents observed data; diamond presents summary operating point; dash line presents 95% confidence contour; points dotted line presents 95% prediction contour.

Ten studies containing 1,446 subjects were summarized to assess the diagnostic accuracy of the combined test of TTF-1 and Napsin A. The pooled sensitivity and specificity were 0.76 (95% CI: 0.69–0.83), and 1.00 (95% CI: 0.92–1.00), respectively. Forest plots showed relative strength of the diagnostic accuracy of the combined test (Figure 3). The positive and negative LRs of the studies were 877.60 (95% CI: 8.40–91533.40) and 0.24 (95% CI: 0.18–0.32), respectively. The pooled diagnostic odds ratio was 3719 (95% CI: 33–414884). The area under the sROC was 0.92 (95% CI: 0.89–0.94), indicating high precision of the combined test (Figure 4).

Significant heterogeneity was observed both in sensitivity (Q = 44.33; *P*≤0.01; I^2^ = 79.70) and specificity (Q = 48.76; *P*≤0.01; I^2^ = 81.54). Subsequently, subgroup analyses and meta-regression were conducted to identify the sources of heterogeneity with respect to the patient's location, sample size, specimen type, and threshold of staining positivity. The results of the subgroup analyses showed significant heterogeneity observed for sensitivity in all subgroups (Table 2). The heterogeneity of specificity in studies conducted in Asians was not statistically significant (I^2^ = 18.84; *P* = 0.30). However, it was adverse in studies conducted in western countries (I^2^ = 87.40; *P*≤0.01). When compared to holistic heterogeneity of specificity (I^2^ = 81.54; *P*≤0.01), the heterogeneity was reduced and was not statistically significant for studies with large sample sizes (I^2^ = 40.08; *P* = 0.14), similar in studies that only contained resected specimens as test materials (I^2^ = 34.35; *P* = 0.17). And the sensitivity and specificity of the seven studies as just resected specimens used were not significant different from that of the ten studies as resected specimens and biopsies combined (0.74 (95%CI: 0.65–0.82) vs. 0.76 (95%CI: 0.69–0.83)), similar in specificity (1.00 (95%CI: 0.92–1.00) vs. 1.00 (95%CI: 0.92–1.00)). It indicated that the three studies [27], [32], [36] using small biopsy or cytology specimens as the reference diagnostic standard were not responsible for influencing heterogeneity in sensitivity and specificity. Through meta-regression, the source of heterogeneity for sensitivity was found to be the patient's location (*P*≤0.01). The source of heterogeneity for specificity was found to be a result of the patient's location, sample size, and threshold of staining positivity (all with *P*-values of less than 0.001). Furthermore, sensitivity analysis showed that removing study of Noh [24] resulted in a wider range in 95%CI of specificity but the specificity was stable (Table S1).

**Table 2:** Subgroup analysis.

Subgroup		SEN (95% CI)	SPE (95% CI)	AUC	I^2^(SEN,95%CI)	I^2^(SPE,95%CI)
Patient's location	Western	0.80 (0.72–0.86)	1.00 (0.09–1.00)	0.90	70.57 (45.64–95.51)	87.40 (78.73–96.06)
	Asian	0.70 (0.56–0.82)	0.99 (0.96–1.00)	—c	85.96 (73.40–98.52)	18.84 (0.00–100.00)
Sample size	Large^a^	0.77 (0.70–0.83)	1.00 (0.82–1.00)	0.92	78.61 (61.77–95.46)	40.08 (0.00–95.51)
	Small^b^	0.76 (0.58–0.88)	1.00 (0.53–1.00)	0.94	85.63 (72.69–98.56)	81.97 (64.83–99.11)
Specimen type	Resected	0.74 (0.65–0.82)	1.00 (0.92–1.00)	0.96	84.18 (73.58–94.78)	34.35 (0.00–90.83)
Staining positive	≥1%^d^	0.76 (0.65–0.84)	1.00 (0.90–1.00)	0.93	83.88 (73.02–94.74)	78.73 (63.35–94.11)

NOTE:

a , the sample size ≧ 100;

b , the sample size <100;

c , the AUC can't be calculated to get a result;

d , cases with more than 1% of tumor cells staining were classified as positive;

SEN, sensitivity; SPE, specificity; AUC, the summary receiver operating characteristics curve; I^2^, I^2^ statistic; 95%CI, 95% confidence interval.

Finally, the funnel plots for the diagnostic value of combined test did not reveal any evidence of obvious asymmetry, with all *P*-values of the Egger's test greater than 0.05 (Figure S1). Therefore, it was determined that publication bias did not have a statistically significant effect in this meta-analysis.

## Discussion

The combined test of TTF-1 and Napsin A has been considered as a promising alternative tool to subclassify NSCLC in clinical practice. However, the sensitivity and specificity of this combined test has varied greatly in previous studies. The present meta-analysis confirmed that the diagnostic value of this combined test was acceptable in distinguishing AC from SQCC, with a relatively high AUC (0.92, 95%CI: 0.89–0.94) and specificity (1.00, 95%CI: 0.92–1.00), and moderate sensitivity (0.76, 95%CI: 0.69–0.83).

In recent years, the need for accurate subclassification of NSCLC has increased, as emerging evidence has suggested that the specific NSCLC subtypes will respond differently to targeted therapies. The current World Health Organization classification of lung cancer has been based almost entirely on the results of H&E staining. Although H&E evaluation could provide sufficient information to classify NSCLC subtypes in many cases, accurate diagnoses may be limited in case of where: only small biopsies or cytology specimens are available, there is poorly differentiated neoplasm, or there is a marked disruption of the histological architecture. What's more, the concordance rates among pathologists often vary significantly. One study showed that concordance rates among pathologists in subclassifying NSCLC by H&E alone were only 81% [38]. As a result of these limitations, several molecular diagnoses have been proposed to subclassify NSCLC in recent years, such as *EGFR* mutations, *K-ras* mutations, *EML4–ALK* fusions and miRNA profiling [11], [39]–[42]. *EGFR* mutations, *K-ras* mutations and *EML4–ALK* fusions have been shown to be primarily restricted to lung adenocarcinoma. Following identification, specific therapies could then be used, such as the EGFR inhibitors Erlotinib and Gefitinib that showed the greatest benefit in EGFR mutation-positive tumors, which were predominantly adenocarcinoma. Similarly, tumors presenting *EML4-ALK* fusion proteins or fusion gene could be treated with greater success by Crizotinib, an inhibitor of anaplastic lymphoma kinase (ALK). Although these molecular diagnostic markers were precise and contributed to targeted therapies, most of the studies used *EGFR* and *K-ras* mutations to classify the subtypes of lung adenocarcinoma. An additional factor that must be considered is that *EML4–ALK* fusions were detected in only about 5% of lung adenocarcinoma, and it was mutually exclusive to other *EGFR* mutations and *K-ras* mutations [43]. These problems were further compounded by the fact that, molecular testing for these markers was complex and not easy to perform. Similar problems were met in miRNA profiling. MiRNA, as noncoding single-stranded RNAs regulating gene expression, were reported as a highly reliable method in distinguishing lung SQCC from AC [11], [13]. However, miRNA profiling had high requirements for specimen preservation conditions as miRNA is easily degraded, which presents a challenge for clinical diagnoses. Compared to these markers, testing for TTF-1 and Napsin A not only had a high diagnostic value, but also was easy to perform. This would be a powerful alternative method to distinguish AC from SQCC in lung cancer diagnosis and treatment.

Heterogeneity was statistically significant for both sensitivity and specificity of the combined detection of TTF-1 and Napsin A. Subgroup analyses showed that the stratification of the confounding factors did not remove the heterogeneity for sensitivity. For specificity however, the heterogeneity was not statistically significant in studies that were conducted in Asians, had large sample sizes and studies in which resected specimens were used. Meta-regression suggested that the patient's location contributed the most to the heterogeneity of sensitivity, and the patient's location, sample size and threshold of staining positivity were the sources of heterogeneity for specificity. The heterogeneity among studies conducted in different locations may be attributed most to ethnic differences, which may lead to different expression levels of TTF-1 and Napsin A. The heterogeneity in specificity among studies with different sample sizes may be due to the fact that the entire spectrum of patients in studies with small sample sizes was not representative in most cases. In order to further explore influence of specimen types on the pool sensitivity and specificity of the combined test, we compared pool sensitivity and specificity of the rest seven studies using resected samples only with those of the ten studies as resected specimens and biopsies combined. The results indicated that the pool sensitivity and specificity of the rest seven studies were not significant different from that of the ten studies (0.74 (95%CI: 0.65–0.82) vs. 0.76 (95%CI: 0.69–0.83) and 1.00 (95%CI: 0.92–1.00) vs. 1.00 (95%CI: 0.92–1.00)), which was consistently found not to be a source of heterogeneity for sensitivity and specificity in meta-regression analysis. Sensitivity analysis showed that the heterogeneity was not statistically significant until the Fatima (2011) study was excluded, supporting the notion that the Fatima (2011) study affected the heterogeneity of specificity the most. This was due to the study's poorly differentiated areas of AC and SQCC.

Although the study provides a comprehensive assessment on the diagnostic value of the combined test of TTF-1 and Napsin A, several precautions should be taken into account when interpreting the results. First, significant inter-study heterogeneity was found. Second, the estimates that we obtained were not adjusted to account for other variables such as tumor size, histologic differentiation, and clinical staging. However, we still observed trends supporting the high diagnostic value of the test in patients with well- and moderately-differentiated lung adenocarcinoma in their tissue sections. Third, population characteristics such as the smoking, sex and age should be put forward in the included studies, so that a more detailed subgroup analyses can be made to explain the heterogeneity.

Overall, our meta-analysis showed that the combined test of TTF-1 and Napsin A was a high diagnosis accuracy alternative diagnostic test in classifying AC and SQCC in NSCLC.

## Supporting Information

Figure S1 **Deeks' funnel Plot Asymmetry test of combined TTF-1 and Napsin A in distinction between AC from SQCC.** Circle presents study; solid line presents regression line. See Figure S1.tif file.
(TIF)

Table S1 Sensitive analysis for studies included in the meta-analysis. See Table S1.doc file.
(DOC)

Checklist S1 **PRISMA 2009 Checklist.**
(DOC)
