# Supplementary figures and images for: The High Diagnostic Accuracy of Combined Test of Thyroid Transcription Factor 1 and Napsin A to Distinguish between Lung Adenocarcinoma and Squamous Cell Carcinoma: A Meta-Analysis
Source: PLoS One. 2014 Jul 8;9(7):e100837. doi: 10.1371/journal.pone.0100837 (PMC4086931; doi:10.1371/journal.pone.0100837)

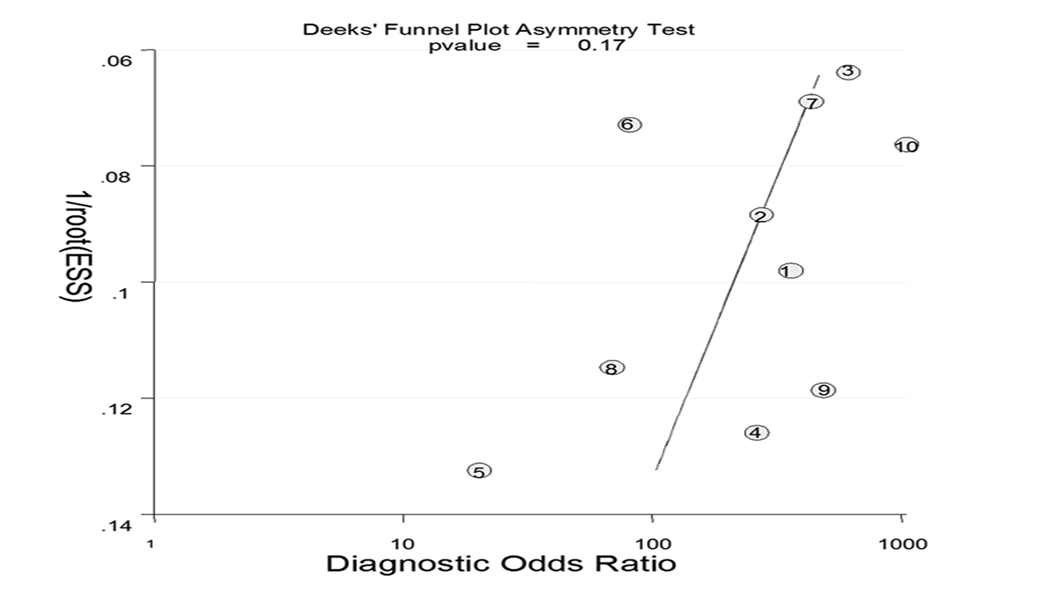

Supplement: Figure S1 — Deeks' funnel Plot Asymmetry test of combined TTF-1 and Napsin A in distinction between AC from SQCC. Circle presents study; solid line presents regression line. See Figure S1.tif file. (TIF) [file pone.0100837.s001.tif]
