# Supplementary material for: The High Diagnostic Accuracy of Combined Test of Thyroid Transcription Factor 1 and Napsin A to Distinguish between Lung Adenocarcinoma and Squamous Cell Carcinoma: A Meta-Analysis
Source: PLoS One. 2014 Jul 8;9(7):e100837. doi: 10.1371/journal.pone.0100837 (PMC4086931; doi:10.1371/journal.pone.0100837)
Supplement: Table S1 — Sensitive analysis for studies included in the meta-analysis. See Table S1.doc file. (DOC) [file pone.0100837.s002.doc]

Table S1. Sensitive analysis for studies included in the meta-analysis.

| **Dropped study** | **SEN ( 95% CI )** | **SPE ( 95% CI )** | **AUC** | **I^2^**(SEN,95%CI) | **I^2^** ( SPE, 95% CI ) |
| --- | --- | --- | --- | --- | --- |
| Bishop | 0.76 ( 0.68 - 0.83 ) | 1.00 ( 0.91 - 1.00 ) | 0.93 | 81.81 ( 70.78 - 92.85 ) | 81.95 ( 71.03 - 92.88 ) |
| Yang | 0.76 ( 0.68 - 0.83 ) | 1.00 ( 0.92 - 1.00 ) | 0.93 | 81.79 ( 70.73 - 92.84 ) | 82.46 ( 71.92 - 93.00 ) |
| Zhang | 0.76 ( 0.68 - 0.83 ) | 1.00 ( 0.91 - 1.00 ) | 0.93 | 81.65 ( 70.49 - 92.81 ) | 80.93 ( 69.22 - 92.65 ) |
| Yanagita | 0.76 ( 0.67 - 0.82 ) | 1.00 ( 0.92 - 1.00 ) | 0.92 | 80.86 ( 69.09 - 92.63 ) | 82.29 ( 71.63 - 92.96 ) |
| Fatima | 0.77 ( 0.69 - 0.83 ) | 1.00 ( 0.91 - 1.00 ) | 0.96 | 82.24 ( 71.53 - 92.95 ) | 30.02 ( 0.00 - 83.90 ) |
| Turner ^a^ | — | — | — | — | — |
| Tacha | 0.77 ( 0.69 - 0.84 ) | 1.00 ( 0.91 - 1.00 ) | 0.93 | 81.00 ( 69.33 - 92.66 ) | 79.90 ( 67.39 - 92.42 ) |
| Noh | 0.78 ( 0.72 - 0.83 ) | 1.00 ( 0.75 - 1.00 ) | 0.90 | 72.27 ( 53.55 - 90.99 ) | 82.06 ( 71.21 - 92.90 ) |
| Collins | 0.75 ( 0.67 - 0.82 ) | 1.00 ( 0.92 - 1.00 ) | 0.91 | 80.04 ( 67.63 - 92.45 ) | 81.74 ( 70.65 - 92.83 ) |
| Brunnstrom | 0.74 ( 0.67 - 0.80 ) | 1.00 ( 0.91 - 1.00 ) | 0.89 | 72.90 ( 54.70 - 91.10 ) | 80.30 ( 68.09 - 92.51 ) |

NOTE: a, parameter can't be calculated when the Turner study was drop; SEN, sensitivity; SPE, specificity; AUC, the summary receiver operating characteristics curve; I^2^, I^2^ statistic; 95%CI, 95% confidence interval.
