# Supplementary material for: The High Diagnostic Accuracy of Combined Test of Thyroid Transcription Factor 1 and Napsin A to Distinguish between Lung Adenocarcinoma and Squamous Cell Carcinoma: A Meta-Analysis
Source: PLoS One. 2014 Jul 8;9(7):e100837. doi: 10.1371/journal.pone.0100837 (PMC4086931; doi:10.1371/journal.pone.0100837)
Supplement: Checklist S1 — PRISMA 2009 Checklist. (DOC) [file pone.0100837.s003.doc]

| **-5Section/topic** | **#** | **Checklist item** | **Reported on page #** |
| --- | --- | --- | --- |
| **TITLE** | | |  |
| Title | 1 | Identify the report as a systematic review, meta-analysis, or both. | **The high** **diagnostic accuracy of** **combined test of Thyroid Transcription Factor 1 and Napsin A to distinguish between lung adenocarcinoma and squamous cell carcinoma: A meta-analysis** |
| **ABSTRACT** | | |  |
| Structured summary | 2 | Provide a structured summary including, as applicable: background; objectives; data sources; study eligibility criteria, participants, and interventions; study appraisal and synthesis methods; results; limitations; conclusions and implications of key findings; systematic review registration number. | **Abstract** |
| **INTRODUCTION** | | |  |
| Rationale | 3 | Describe the rationale for the review in the context of what is already known. | The first paragraph of **Introduction** |
| Objectives | 4 | Provide an explicit statement of questions being addressed with reference to participants, interventions, comparisons, outcomes, and study design (PICOS). | The second paragraph of **Introduction** |
| **METHODS** | | |  |
| Protocol and registration | 5 | Indicate if a review protocol exists, if and where it can be accessed (e.g., Web address), and, if available, provide registration information including registration number. | **Literature search in Material and Methods** |
| Eligibility criteria | 6 | Specify study characteristics (e.g., PICOS, length of follow-up) and report characteristics (e.g., years considered, language, publication status) used as criteria for eligibility, giving rationale. | **Selection criteria in Material and Methods** |
| Information sources | 7 | Describe all information sources (e.g., databases with dates of coverage, contact with study authors to identify additional studies) in the search and date last searched. | **Literature search in Material and Methods** |
| Search | 8 | Present full electronic search strategy for at least one database, including any limits used, such that it could be repeated. | **Literature search in Material and Methods** |
| Study selection | 9 | State the process for selecting studies (i.e., screening, eligibility, included in systematic review, and, if applicable, included in the meta-analysis). | **Selection criteria in Material and Methods** |
| Data collection process | 10 | Describe method of data extraction from reports (e.g., piloted forms, independently, in duplicate) and any processes for obtaining and confirming data from investigators. | **Data extraction and quality assessment in Material and Methods** |
| Data items | 11 | List and define all variables for which data were sought (e.g., PICOS, funding sources) and any assumptions and simplifications made. | **Data extraction and quality assessment in Material and Methods** |
| Risk of bias in individual studies | 12 | Describe methods used for assessing risk of bias of individual studies (including specification of whether this was done at the study or outcome level), and how this information is to be used in any data synthesis. | The first paragraph of **Statistical analysis in Material and Methods** |
| Summary measures | 13 | State the principal summary measures (e.g., risk ratio, difference in means). | The first paragraph of **Statistical analysis in Material and Methods** |
| Synthesis of results | 14 | Describe the methods of handling data and combining results of studies, if done, including measures of consistency (e.g., I^2^) for each meta-analysis. | The first paragraph of **Statistical analysis in Material and Methods** |

Page 1 of 2

| **Section/topic** | **#** | **Checklist item** | **Reported on page #** |
| --- | --- | --- | --- |
| Risk of bias across studies | 15 | Specify any assessment of risk of bias that may affect the cumulative evidence (e.g., publication bias, selective reporting within studies). | The second paragraph of **Statistical analysis in Material and Methods** |
| Additional analyses | 16 | Describe methods of additional analyses (e.g., sensitivity or subgroup analyses, meta-regression), if done, indicating which were pre-specified. | The first paragraph of **Statistical analysis in Material and Methods** |
| **RESULTS** | | |  |
| Study selection | 17 | Give numbers of studies screened, assessed for eligibility, and included in the review, with reasons for exclusions at each stage, ideally with a flow diagram. | The first paragraph of **Characteristics and methodological quality of include studies in Results, Figure 1** |
| Study characteristics | 18 | For each study, present characteristics for which data were extracted (e.g., study size, PICOS, follow-up period) and provide the citations. | **Table 1** |
| Risk of bias within studies | 19 | Present data on risk of bias of each study and, if available, any outcome level assessment (see item 12). | The second paragraph of **Characteristics and methodological quality of include studies in Results, Figure 2** |
| Results of individual studies | 20 | For all outcomes considered (benefits or harms), present, for each study: (a) simple summary data for each intervention group (b) effect estimates and confidence intervals, ideally with a forest plot. | **Figure 3, Table 2** |
| Synthesis of results | 21 | Present results of each meta-analysis done, including confidence intervals and measures of consistency. | The first paragraph of **Meta-analysis and heterogeneity in Results, Figure 4** |
| Risk of bias across studies | 22 | Present results of any assessment of risk of bias across studies (see Item 15). | The second and third paragraph of **Meta-analysis and heterogeneity in Results, Figure S1** |
| Additional analysis | 23 | Give results of additional analyses, if done (e.g., sensitivity or subgroup analyses, meta-regression [see Item 16]). | The second paragraph of **Meta-analysis and heterogeneity in Results,** **Table 2, Table S1** |
| **DISCUSSION** | | |  |
| Summary of evidence | 24 | Summarize the main findings including the strength of evidence for each main outcome; consider their relevance to key groups (e.g., healthcare providers, users, and policy makers). | The first three paragraphs of **Discussion** |
| Limitations | 25 | Discuss limitations at study and outcome level (e.g., risk of bias), and at review-level (e.g., incomplete retrieval of identified research, reporting bias). | The fourth paragraph of **Discussion** |
| Conclusions | 26 | Provide a general interpretation of the results in the context of other evidence, and implications for future research. | The fifth paragraph of **Discussion** |
| **FUNDING** | | |  |
| Funding | 27 | Describe sources of funding for the systematic review and other support (e.g., supply of data); role of funders for the systematic review. | Page 2: Funding: This work was supported by the National Science Foundation of China (NSF 81172754 and NSF 81172752). The funders had no role in study design, data collection and analysis, decision to publish, or preparation of the manuscript. |

*From:*  Moher D, Liberati A, Tetzlaff J, Altman DG, The PRISMA Group (2009). Preferred Reporting Items for Systematic Reviews and Meta-Analyses: The PRISMA Statement. PLoS Med 6(6): e1000097. doi:10.1371/journal.pmed1000097

For more information, visit: **www.prisma-statement.org**.

Page 2 of 2
